# Awareness of Dystonic Posture in Patients With Cervical Dystonia

**DOI:** 10.3389/fpsyg.2020.01434

**Published:** 2020-06-23

**Authors:** Gina Ferrazzano, Isabella Berardelli, Daniele Belvisi, Maria Ilenia De Bartolo, Antonella Di Vita, Antonella Conte, Giovanni Fabbrini

**Affiliations:** ^1^Department of Human Neurosciences, Sapienza University of Rome, Rome, Italy; ^2^Department of Neurosciences, Mental Health and Sensory Organs, Suicide Prevention Center, Sant’Andrea Hospital, Sapienza University of Rome, Rome, Italy; ^3^IRCCS NEUROMED, Pozzilli, Italy

**Keywords:** cervical dystonia, dystonic tremor, bodily awareness, proprioceptive dysfunction, sensorimotor integration

## Abstract

**Background:**

Cervical dystonia (CD) is a focal dystonia characterized by sensorimotor integration abnormalities and proprioceptive dysfunction. Since proprioception is essential for bodily awareness, we hypothesized that CD patients may have an impairment in dystonic posture awareness. More information on this issue could be useful to better understand whether dystonic posture affects bodily perception in CD and could help in the development of specific rehabilitation strategies based on proprioceptive input manipulation to restore bodily awareness.

**Objectives:**

The aim of our study was to investigate dystonic posture and head tremor awareness in CD patients by comparing evaluations performed by CD patients with those performed by a neurologist expert in movement disorders.

**Methods:**

We enrolled 25 CD patients. We investigated dystonic posture and head tremor awareness in CD patients using a standardized protocol in which patients were asked to describe the type of dystonic pattern, both while viewing standardized images of different CD subtypes (torticollis, laterocollis, anterocollis, and retrocollis) and after watching a video recording of their dystonic posture and head tremor.

**Results:**

We found that 72% of CD patients correctly recognized their dystonic posture when viewing standardized images, whereas 84% of CD patients were able to identify their dystonic pattern when watching a video recording of themselves. CD patients also displayed a preserved awareness of their head tremor. We did not find any associations between dystonic pattern awareness and clinical or demographic features.

**Conclusion:**

Contrary to our hypothesis, the majority of CD patients have a preserved awareness of their dystonic pattern and tremor.

## Introduction

Cervical dystonia (CD) is a form of focal dystonia characterized by different patterns of abnormal postures such as torticollis, laterocollis, anterocollis, and retrocollis, which may occur separately or in combination (Chan et al., 1991; Jankovic et al., 1991). Head tremor is also frequently observed in CD patients (Defazio et al., 2013, 2015; Hvizdošová et al., 2020). Several neurophysiological investigations have reported that CD patients have abnormalities in sensorimotor integration and proprioceptive input processing (Abbruzzese and Berardelli, 2003; Quartarone and Hallett, 2013; Avanzino and Fiorio, 2014; Conte et al., 2019). Furthermore, there is evidence that manipulation of proprioceptive information through vibration, kinesiotaping, and transcutaneous electrical nerve stimulation (TENS) improves dystonic symptoms (Karnath et al., 2000; Tinazzi et al., 2005, 2006; Pelosin et al., 2013; Avanzino and Fiorio, 2014). Proprioceptive input processing is essential for bodily awareness (Proske and Gandevia, 2012). Bodily awareness may be considered the internal understanding of the body that involves the correct elaboration of body schema, i.e., the sensorimotor representation of the body, and body image, i.e., the construction of bodily perception that involves both sensory information and cognitive processes (Vignemont, 2010). Proprioception and bodily awareness are therefore closely interconnected and both have a crucial role in motor control. Due to current evidence that CD patients have abnormal proprioceptive processing associated with their dystonic posture and the presence of tremor, we hypothesized that CD patients would also have impaired awareness of their altered posture. Since vibration-induced proprioceptive input manipulation transiently modulates dystonic posture (Karnath et al., 2000; Avanzino and Fiorio, 2014), a better understanding of whether bodily awareness is altered in CD patients may prompt future studies to investigate rehabilitative strategies based on proprioceptive input manipulation.

For this purpose, in this pilot investigation, we investigated dystonic posture and head tremor awareness in CD patients by means of a standardized protocol in which patients were asked to describe the type of dystonic pattern, both while viewing standardized images of different CD subtypes and after watching a video recording of their dystonic posture. We then compared the evaluations performed by CD patients with those performed by a neurologist expert in movement disorders.

## Materials and Methods

### Study Participants and Clinical Assessment

A total of 25 CD patients (6 males, mean age 55 ± 11 years) were enrolled from the movement disorders outpatient clinic of the Department of Human Neurosciences, Sapienza University of Rome. CD diagnosis was based on standardized clinical criteria (Jinnah et al., 2013). Exclusion criteria included any neurological abnormalities other than tremor, clinically significant cognitive deficits as determined by a score <26 on the Montreal Cognitive Assessment (MoCA) (Nasreddine et al., 2005), or clinically significant depressive or anxiety symptoms as assessed by the Hamilton Depression Rating Scale (HAM-D) (Hamilton, 1960) and the Hamilton Anxiety Rating Scale (HAM-A) (Hamilton, 1959). CD severity was assessed according to the Toronto Western Spasmodic Torticollis Rating Scale (TWSTRS) (Comella et al., 1997). To exclude any possible confounding due to botulinum toxin effect, all CD patients were evaluated about 4 months after the last botulinum toxin injection. The experimental procedure was conducted in accordance with the Declaration of Helsinki and was approved by the local institutional review board. All participants signed a written informed consent form.

### Video Protocol

All CD patients underwent a standardized video recording obtained in controlled conditions in a quiet room with artificial light. We focused the camera on the upper half of the body with the patient seated in a comfortable position. Patients were first recorded at rest for 10 s and then during voluntary movements of right and left rotation and flexion and extension of the head. CD patients were also recorded during a back-and-forth walk of 10 m.

### Training to Recognize Dystonic Postures

All CD patients were trained on the different possible clinical patterns of CD. For this purpose, we showed patients images taken from an atlas that provided illustrations on all possible CD patterns (torticollis, laterocollis, anterocollis, retrocollis) (Jost, 2009). During the training, we showed and described to patients the images representative of the four single CD patterns, namely torticollis, laterocollis, retrocollis, and anterocollis, as well as the main combinations of these patterns. Images were presented one at a time. The associated pattern name was displayed below the image. The training lasted about 5 min for each CD patient. We ensured that patients had learned the procedure before moving to the next part of the study.

### Study Paradigm

After the training session described above, each CD patient was asked to define his/her own dystonic pattern by selecting the corresponding image in the atlas (Jost, 2009). For patients with a combined pattern of dystonia, we asked the patient to describe the predominant dystonic pattern (i.e., torticollis, laterocollis, retrocollis, anterocollis). We also asked whether they were able to recognize the presence of tremor.

Patients were subsequently shown videos of themselves that were previously recorded according to the standardized video protocol and were again asked to identify their dystonic pattern in the atlas (Jost, 2009). Each patient was also asked to recognize the presence or absence of head tremor. The report of each CD patient was compared to the clinical assessment performed by a neurologist expert in movement disorders (GF).

### Statistical Analysis

Data are expressed as mean ± standard deviation (SD) unless otherwise indicated. Statistical analysis was performed using SPSS software, version 25. Kendall rank correlation coefficient was used to evaluate the concordance between CD patient evaluations of dystonic pattern and those performed by the neurologist. Demographic and clinical features of patients with preserved awareness were compared with those of patients who had altered awareness by using the Mann-Whitney *U* test, one-way analysis of variance (ANOVA), and student’s *t*-test as appropriate. Spearman’s correlation coefficient was used to investigate the relationship between dystonic pattern awareness and clinical and demographic parameters. Linear regression analysis was used to identify demographic and clinical factors possibly associated with dystonic pattern awareness. A *p* value <0.05 indicated statistical significance. Bonferroni’s correction for multiple comparisons was applied.

## Results

Demographic and clinical features of CD patients are shown in Table 1.

**TABLE 1:** Clinical and demographic data of CD patients.

Number of patients	Age range (y)	Disease duration (y)	TWSRS total score	Dystonic pattern	Head tremor
7	30–45	9.1 ± 7.5	15.4 ± 9.7	1 L/R; 2L; 3 L/T; 1 T	2/7
14	46–61	14.5 ± 9.8	19.2 ± 11.4	3 L; 4L/T; 2 L/R; 4 T; 1 T/L	6/14
4	62–76	10.2 ± 6.9	12.5 ± 4.0	2 L; 1 L/T; 1 T/R	1/4

*Data are expressed as mean ± standard deviation and as number of CD patients. Y: years; TWSTRS: Toronto Western Spasmodic Torticollis Rating Scale; L: laterocollis; T: torticollis; R: retrocollis.*

### Awareness of Dystonic Pattern

Kendall rank correlation coefficient showed a significant concordance between CD patient evaluations of dystonic patterns and those performed by the neurologist, both while viewing standardized images (*r* = 0.61; *p* = 0.0002, *p* < 0.025 indicated statistical significance after Bonferroni’s correction) and video recordings (*r* = 0.62; *p* = 0.0003, *p* < 0.025 indicated statistical significance after Bonferroni’s correction). Approximately 18 of the 25 CD patients (72%) recognized their dystonic pattern when viewing a standardized image and 21 (84%) recognized their dystonic pattern when watching the video recording. Of the nine CD patients with head tremor, seven recognized this feature when watching their video recording. Patients who had preserved awareness had similar demographic and clinical features to patients with altered awareness (all *p* values >0.05).

### Relationship Between Dystonic Pattern Awareness and Clinical and Demographic Variables

No significant correlations emerged between dystonic posture awareness and clinical or demographic features, including gender, age, age at onset, disease duration, and disease severity as tested by TWSTRS (all *p* values >0.05). Similarly, linear regression analysis did not show any significant associations between dystonic posture awareness and demographic or clinical variables (all *p* values >0.05).

## Discussion

This pilot study showed that 72% of CD patients correctly recognized their dystonic posture when comparing their pattern with that shown on a standardized image. Similarly, 84% of CD patients were able to identify their dystonic pattern when watching a video recording of themselves. CD patients also displayed a preserved awareness of their head tremor. Finally, we did not find any association between dystonic pattern awareness and clinical or demographic features.

We took several precautions to ensure that the data we gathered were reliable. We did not include patients with significant cognitive or emotional disturbances that might have influenced dystonic pattern awareness. We showed patients standardized, representative, and simple images of the different CD phenotypes, and video recordings were performed according to a standardized procedure used in international studies. In addition, since botulinum toxin injection improves dystonic posture and tremor by changing proprioceptive input, we tested patients at least 4 months after the last botulinum toxin injection.

Based on previous studies showing that patients with different movement disorders such as Parkinson’s disease and Huntington’s disease have altered movement disorder awareness (Snowden et al., 1998; Vitale et al., 2001; Hoth et al., 2007; Sitek et al., 2011; Pietracupa et al., 2013), we expected that CD patients would also have altered dystonic posture and head tremor awareness. This hypothesis was also substantiated by neurophysiological studies showing that CD patients have abnormal proprioceptive processing. Unexpectedly, we found that the majority of CD patients were able to correctly identify their pattern of dystonic posture and the presence of head tremor.

To understand why bodily awareness, in terms of dystonic posture and tremor, is preserved in CD patients, it may be useful to compare mechanisms underlying bodily awareness with those involved in CD pathophysiology. Bodily awareness is based on two distinct and interrelated components that include sensorimotor integration mechanisms: (1) body schema, the sensorimotor representation of the body that guides movements, and (2) body image, the construction of bodily perception that involves sensory information and cognitive processes (Vignemont, 2010). Although the presence of dystonic posture and head tremor determines aberrant proprioception arriving at somatosensory brain areas that are also involved in bodily awareness (Holmes and Spence, 2004; Mooshagian et al., 2008; Berlucchi and Aglioti, 2010; Pietrini et al., 2010), this proprioceptive overflow apparently leaves cortical processes that allow the construction of body schema and body image unaffected. We may therefore speculate that bodily awareness is normal in the majority of CD patients because proprioceptive information is normally conveyed or even prioritized (Conte et al., 2018) from among the various signals arriving at the cortical level. Conversely, in the subgroup of patients who did not show dystonic posture awareness it could be hypothesized that network derangement (Quartarone and Hallett, 2013; Prudente et al., 2014; Jinnah and Hess, 2018; Schirinzi et al., 2018; Conte et al., 2019; Corp et al., 2019) extends to brain regions involved in bodily awareness that are not primarily affected by dystonia.

Our findings that awareness does not correlate with disease severity or duration may suggest that dystonic awareness does not depend on processes that deteriorate gradually as the disease progresses. However, the lack of correlation between clinical features and awareness may also be due to the limited sample size. Another limitation of our study is the absence of a control group of patients with abnormal posture due to non-dystonic conditions, which would have allowed us to compare evaluation accuracy between these different patient groups. However, we compared CD patient self-evaluations with evaluations performed by a neurologist expert in movement disorders and the high level of agreement between CD patient self-evaluation and expert evaluation allowed us to conclude that the majority of CD patients accurately recognized their main dystonic pattern.

In conclusion, our findings show that unlike patients affected by other hyperkinetic movement disorders (i.e., levodopa-induced dyskinesia in Parkinson’s disease and Huntington’s disease), the majority of CD patients have a preserved awareness of their dystonic pattern and tremor. Future studies specifically investigating body schema and body image mechanisms in larger samples of patients with different types of dystonia are needed to draw definite conclusions on bodily awareness in dystonia.

## Data Availability Statement

The datasets generated for this study are available on request to the corresponding author.

## Ethics Statement

The studies involving human participants were reviewed and approved by Comitato Etico, Azienda Ospedaliera Universitaria Policlinico Umberto I, Viale del Policlinico, 155, 00161 Rome, Italy. The patients/participants provided their written informed consent to participate in this study.

## Author Contributions

GFe, AC, and GFa: conception and design of the study. GFe, DB, and AC: analysis and interpretation of data. GFe, IB, and MD: acquisition of data. GFe, AD, AC, and GFa: drafting and critically revising the manuscript. GFe, IB, DB, MD, AD, AC, and GFa: final approval of the version to be submitted. All authors contributed to the article and approved the submitted version.

## Conflict of Interest

The authors declare that the research was conducted in the absence of any commercial or financial relationships that could be construed as a potential conflict of interest.
